# Properly Substituted Analogues of BIX-01294 Lose Inhibition of G9a Histone Methyltransferase and Gain Selective Anti-DNA Methyltransferase 3A Activity

**DOI:** 10.1371/journal.pone.0096941

**Published:** 2014-05-08

**Authors:** Dante Rotili, Domenico Tarantino, Biagina Marrocco, Christina Gros, Véronique Masson, Valérie Poughon, Fréderic Ausseil, Yanqi Chang, Donatella Labella, Sandro Cosconati, Salvatore Di Maro, Ettore Novellino, Michael Schnekenburger, Cindy Grandjenette, Celine Bouvy, Marc Diederich, Xiaodong Cheng, Paola B. Arimondo, Antonello Mai

**Affiliations:** 1 Dipartimento di Chimica e Tecnologie del Farmaco, Sapienza Università di Roma, Roma, IT; 2 USR3388 CNRS-Pierre Fabre ETaC, CRDPF, Toulouse, France; 3 Department of Biochemistry, Emory University School of Medicine, Atlanta, Georgia, United States of America; 4 DiSTABiF, Seconda Università di Napoli, Caserta, Italy; 5 Dipartimento di Farmacia, Università di Napoli “Federico II”, Napoli, IT; 6 Laboratoire de Biologie Moléculaire et Cellulaire du Cancer (LBMCC), Luxembourg, Luxembourg; 7 Department of Pharmacy, College of Pharmacy, Seoul National University, Seoul, Korea; 8 Istituto Pasteur-Fondazione Cenci Bolognetti, Sapienza Università di Roma, Roma, IT; Albert-Ludwigs-University, Germany

## Abstract

Chemical manipulations performed on the histone H3 lysine 9 methyltransferases (G9a/GLP) inhibitor BIX-01294 afforded novel desmethoxyquinazolines able to inhibit the DNA methyltransferase DNMT3A at low micromolar levels without any significant inhibition of DNMT1 and G9a. In KG-1 cells such compounds, when tested at sub-toxic doses, induced the luciferase re-expression in a stable construct controlled by a cytomegalovirus (CMV) promoter silenced by methylation (CMV-luc assay). Finally, in human lymphoma U-937 and RAJI cells, the *N*-(1-benzylpiperidin-4-yl)-2-(4-phenylpiperazin-1-yl)quinazolin-4-amine induced the highest proliferation arrest and cell death induction starting from 10 µM, in agreement with its DNMT3A inhibitory potency.

## Introduction

Epigenetics studies all regulatory mechanisms involving heritable changes in gene expression without alteration of the DNA coding sequence, determining cell fate and phenotype [1]–[5]. Among the epigenetic signals, the DNA methylation occurs at the C5 position of the cytosine ring mostly in a CpG dinucleotide context, through the action of three active DNA methyltransferases (DNMTs): DNMT1, DNMT3A and DNMT3B that catalyze the transfer of a methyl group from *S*-adenosyl-l-methionine (AdoMet) to the C5-cytosine [6]. In addition, another related protein lacking catalytic activity, DNMT3L, physically interacts and stimulates enzymatic activities of DNMT3A or DNMT3B [7]. DNMT1 is responsible for the maintenance of methylation patterns during cell division, with a preference for hemimethylated CpG dinucleotides, and performs a crucial role in normal mammalian development, cell proliferation and survival. DNMT3A and DNMT3B are de novo methyltransferases, adding methyl groups in unmethylated genomic sequences at CpG dinucleotides, and are highly expressed in early embryonic cells, downregulated after differentiation and in adult somatic tissues, and overexpressed in cancer [1], [6].

**Figure 1:**
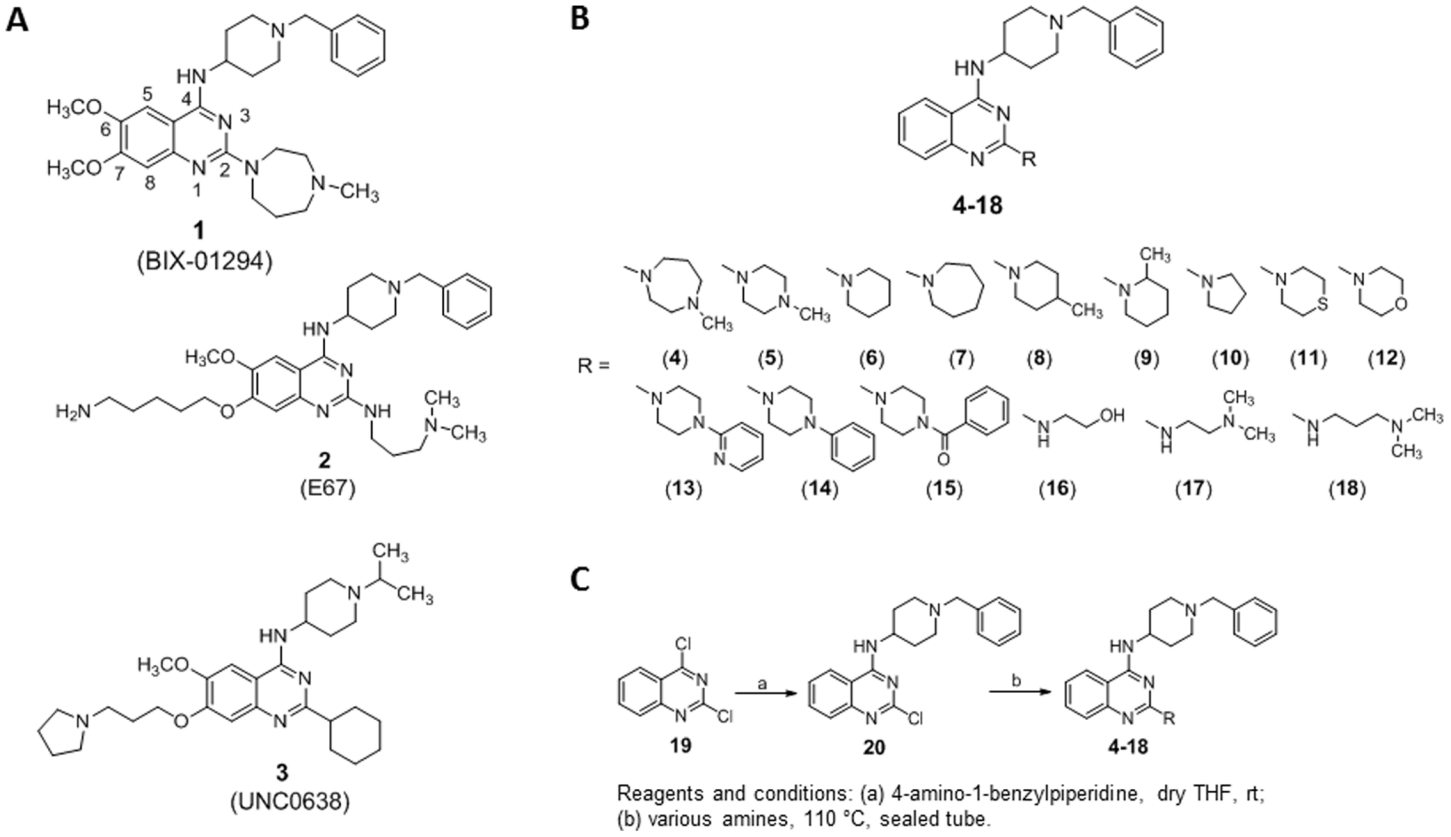
Quinazoline-based modulators of epigenetic targets. (A) Known quinazolines (**1–3**) as G9a/GLP inhibitors. (B) Novel 6,7-desmethoxyquinazolines **4–18**. (C) Synthetic scheme for the preparation of **4–18**.

During our investigation of small molecule epigenetic modulators, we described various series of compounds able to inhibit protein/histone methyltransferases, a subfamily of AdoMet-dependent methyltransferases catalyzing the (poly)methylation of arginine (Arg) or lysine (Lys) residues in both histone and non-histone proteins [8]–[13]. In particular, chemical manipulation of BIX-01294 (**1**) [14] (Figure 1A), a specific G9a/G9a-like protein (GLP) histone H3 Lys9 (H3K9) methyltransferase inhibitor, led to E67 (**2**) [15] (Figure 1A), bearing a 5-aminopentyloxy substituent at the C7 position of the quinazoline ring with enhanced G9a/GLP inhibitory activity in vitro and reduced toxicity in vivo. X-ray crystal structures of **1** and **2** complexed with GLP [15], [16] showed that for both compounds the quinazoline scaffold, linked to the C4-(1-benzyl-4-piperidinylamino) and the C2-diazepane (**1**) or C2-(3-dimethylaminopropylamino) (**2**) moieties, resembles the bound conformation of histone H3 Lys4 to Arg8, while the C7-(5-aminopentyloxy) chain of **2** mimics the target Lys9 of histone H3 in the substrate peptide binding groove. For both compounds, the C6-methoxy group is inserted into a shallow surface pocket mimicking the H3A7 side chain and is crucial for binding to H3K9 modification enzymes (methyltransferases as well as demethylases), discriminating them from other enzymes, such as for example H3K4 methyltransferases and demethylases [15]–[17].

Chemical changes performed by other authors on **1** led to UNC0638 (**3**) [18] (Figure 1A), a potent and selective G9a/GLP inhibitor featuring the 1-*iso*-propyl-4-piperidinylamino moiety at C4, the cyclohexyl group at C2, the 3-(1-pyrrolidino)propyloxy chain at C7 and the methoxy group at C6 position of the quinazoline ring. Compound **3** showed nanomolar potency against G9a, excellent cell permeability and robust on-target activity in cells [18]. Interestingly, compound **3** was found to be inactive against other histone lysine methyltransferases, but displayed high micromolar activity against DNMT1 [18]. This observation suggested that the quinazoline moiety, used to develop lysine methyltransferase and demethylase inhibitors, could also be suitable to design, with appropriate substitutions, compounds able to inhibit DNMTs. In addition, we thought that such quinazoline-based compounds lacking the C6 methoxy group should not be able to bind H3K9 modifying enzymes. Thus, we prepared the series of 6,7-desmethoxy quinazolines **4–18** (Figure 1B) by maintaining the 1-benzyl-4-piperidinylamino moiety (typical of **1** and **2**) at the C4 position of the quinazoline ring and changing the substituent at C2, spanning from differently sized cyclic amines (containing or not heteroatoms and/or lipophilic groups) to open-chain amines.

Here we report the evaluation of the quinazolines **4–18** against human DNMT1 (hDNMT1) and the C-terminal catalytic domain of human DNMT3A (hDNMT3A). For selected derivatives, their effect on the H3K9 methyltransferase G9a was also assessed. Some quinazolines were then tested in a cytomegalovirus-luciferase (CMV-luc) construct in KG-1 cells, repressed by methylation, in order to detect their capability to reactivate gene expression in cells [19]. Docking studies have been performed to gain insight on the binding mode of the most potent compounds on hDNMT3A. Finally, selected derivatives were tested in human leukemia U-937 and in Burkitt’s lymphoma RAJI cells, at 1–10–100 µM from 24 to 72 h, to determine their effects on proliferation and viability.

## Materials and Methods

### Chemistry

Melting points were determined on a Buchi 530 melting point apparatus and are uncorrected. NMR spectra were recorded at 400 MHz (1H) and 100 MHz (13C) on a Bruker AC 400 spectrometer; chemical shifts are reported in δ (ppm) units relative to the internal reference tetramethylsilane (Me_4_Si). All compounds were routinely checked by TLC and ^1^H-NMR. TLC was performed on aluminium-backed silica gel plates (Merck DC, Alufolien Kieselgel 60 F_254_) with spots visualized by UV light. All solvents were reagent grade and, when necessary, were purified and dried by standard methods. Concentration of solutions after reactions and extractions involved the use of a rotary evaporator operating at reduced pressure of ∼ 20 Torr. Organic solutions were dried over anhydrous sodium sulphate. Elemental analysis has been used to determine purity of the described compounds, that is >95%. Analytical results are within ±0.4% of the theoretical values (see Table S1 in File S1). All chemicals were purchased from Aldrich Chimica, Milan (Italy), or from Lancaster Synthesis GmbH, Milan (Italy), and were of the highest purity.

Preparation of *N*-(1-benzylpiperidin-4-yl)-2-chloroquinazolin-4-amine (**20**). See File S1.

General procedure for the preparation of 4-(1-benzylpiperidin-4-yl)-2-(cyclo/alkylamino)quinazolin-4-amines (**4–18**). Example: *N*-(1-benzylpiperidin-4-yl)-2-morpholinoquinazolin-4-amine (**12**). A mixture of *N*-(1-benzylpiperidin-4-yl)-2-chloro-quinazolin-4-amine **20** (1 eq, 0.6 mmol, 0.2 g) and morpholine (5 eq, 2.8 mmol, 0.25 mL) was placed in a sealed tube and stirred at 110°C for 1 h. After cooling to room temperature, 5 mL of water were added and the resulting precipitate was filtered and washed with water. The obtained solid residue was recrystallized from petroleum ether to provide pure **12** as a white solid. Mp: 110–112°C (petroleum ether). Yield: 70%. ^1^H-NMR (CDCl_3_): δ 1.71–1.76 (m, 2H, C*H*-piperidine ring), 2.14–2.31 (m, 4H, C*H*-piperidine ring), 2.96 (m, 2H, C*H*-piperidine ring), 3.61 (s, 2H, C*H_2_*Ph), 3.80–3.87 (m, 8H, C*H*-morpholine ring), 4.18 (m, 1H, NHC_4_-*H*-piperidine), 5.56 (d, 1H, N*H*), 7.11 (m, 1H, C_6_-*H* quinazoline ring), 7.31–7.38 (m, 5H, C-*H* phenyl ring), 7.50–7.53 (m, 3H, C_5,7,8_-*H* quinazoline ring). ^13^C-NMR (CDCl_3_) δ 30.3, 48.7, 51.9, 56.8, 64.7, 66.3, 110.3, 123.0, 127.2, 127.7, 127.8, 128.4, 128.8, 132.7, 138.6, 152.3, 160.1, 184.6 ppm. HR-MS (ESI) calculated for C_24_H_30_N_5_O [M+H]^+^, 404.2450; found, 404.2446.

Chemical and physical data for compounds **4–11**, **13–18** are reported in File S1.

### DNMT1 Assay

His-DNMT1 (182 kDa, human) was cloned, expressed and purified as described by Lee *et al*. [20]. The DNMT1 assay was performed according to Gros *et al*. [21]. Briefly, the reaction was started by addition of 90 nM of DNMT1 on a mix containing the tested compound (up to 1% DMSO), 1 µM of a AdoMet/[methyl-^3^H]-AdoMet mix in a ratio of 3-to-1 (isotopic dilution 1*:3) and 0.3 µM of biotinylated DNA duplex in 10 µL final volume. The reaction was incubated at 37°C for 2 h in reaction buffer (20 mM HEPES pH 7.2, 1 mM EDTA, 50 mM KCl, 25 µg/mL BSA). 8 µL are then transferred into a streptavidin-coated Flashplate PLUS (PerkinElmer) containing 190 µL of 20 µM AdoHcy (Sigma-Aldrich) in 50 mM Tris-HCl pH 7.4. The Flashplate was agitated at room temperature for 1 h, washed three times with 200 µL of 0.05% Tween-20 in 50 mM Tris-HCl pH 7.4 and read in 200 µL of 50 mM Tris-HCl pH 7.4 on TopCount NXT (PerkinElmer). Percentages of inhibition were calculated with the same formula as in DNMT3A assay.

### DNMT3A Assay

DNMT3A enzyme inhibition was adapted from the restriction-based fluorescence assay protocol described in Ceccaldi *et al.* [22]. Briefly, a 5'-labelled biotin oligonucleotide is hybridized to its complementary strand labelled with 6-carboxyfluorescein at the 3′-end into a 384 well microplate (black Optiplates; Perkin Elmer) pre-coated with avidin. The duplex contains a unique CpG site overlapping with a restriction site of a methylation sensitive restriction enzyme. The C-terminal catalytic domain of human DNMT3A (residues 623–908), produced as described [7], was added in each well (200 ng/well) and mixed with the chemical compounds at desired concentrations and freshly prepared AdoMet (20 µM final concentration) to start the reaction in a total volume of 50 µL. After 1 hour incubation at 37°C each well were washed three times with PBS, 0.05% Tween-20, 500 mM NaCl and three more times with PBST. Specific fluorescent signals were detected with the methylation-sensitive restriction enzyme HpyCH4IV (NEB) as described and measured on a Perkin Elmer Envision detector. The percentage of inhibition is reported. The formula used to calculate the percentage of inhibition is [(X-Y)/X]×100, where X is the signal determined in the absence of the inhibitor and Y is the signal obtained in the presence of the inhibitor. The concentration at which 50% of efficacy of inhibition is observed (EC_50_) was determined by analysis of a concentration range of the tested compound in triplicates. The non-linear regression fittings with sigmoidal dose-response (variable slope) were performed with GraphPad Prism 4.03 (GraphPad Software).

### CMV-luc Assay

KG-1 cell line, stably transfected with the luciferase Firefly (Luc+ from pGL3 by Promega) reporter gene under the control of the CMV promoter (from pEGFP-N1 by Clontech) partially methylated (50%), is seeded at 20,000 cell per well in 96-well plate. After 24 h of incubation in the presence of the compounds or the solvent DMSO, the induction of the promoter is measured by quantification of luciferase with the Brite-lite assay system (Perkin Elmer) according to the manufacturer protocol. The luminescence is measured on *EnVision* Multilabel Plate Reader (Perkin Elmer) and the data are expressed in induction factor compared to the DMSO control condition. The mean of 2–4 experiments and its standard error is reported.

### G9a Assay

Human G9a (residues 786–121 0; accession II NM_006709) was expressed as *N*-terminal GST fusion protein in *E. coli*. The reaction buffer was 50 mM Tris-HCl, pH 8.5, 50 mM NaCl, 5 mM MgCl_2_, 1 mM dithiothreitol (DTT), 1 mM PMSF, and 1% DMSO. Standard substrate concentrations were 5 µM biotinylated H3 (1–21) peptide (AnaSpec) substrate and 0.1 mM AdoMet. For control compound IC_50_ determinations, the test compounds were diluted in DMSO and then added to the enzyme/substrate mixture in nanoliter amounts by using an acoustic technology (Echo 500; Lab-cyte). The reaction was initiated by the addition of ^3^H-AdoMet, and incubated at 30°C for 1 h. The reaction was detected by a filter-binding method. Data analysis was performed using Graph Pad Prism software for curve fits, and GraFit (Erithacus) for global fit of kinetic studies. To determine the effects of **4**, **10**, **13** and **14** against G9a, the quinazoline compounds were tested in a 10-dose IC_50_ mode with 2-fold serial dilution starting at 400 µM. The results are summarized in Table S2 in File S1.

### Docking Studies

Prior to docking calculations, the Epik software was used to calculate the most relevant ionization and tautomeric state of compounds **4** and **14** [23]. Then the Glide program of the Schrodinger package [24] was used to dock **4** and **14** to the DNMT3A structure (PDB 2QRV). The receptor grid generation was performed for the box with a center in the putative binding site. The size of the box was determined automatically. The extra precision mode (XP) of Glide was used for docking. The ligand scaling factor was set to 1.0. The geometry of the ligand binding site of the complex between 10 and the receptor was then optimized. The binding site was defined as 10 and all amino acid residues located within 8 Å from the ligand. All the receptor residues located within 2 Å from the binding site were used as a shell. The OPLS2005 force field was used for energy minimization. Water was used as an implicit solvent, and a maximum of 5000 iterations of the Polak–Ribier conjugate gradient minimization method was used with a convergence threshold of 0.01 kJ mol^–1^ Å^–1^. All complex pictures were rendered employing the UCSF Chimera software [25].

### Cellular Assays

U-937 and RAJI cell lines were purchased from Deutsche Sammlung für Mikroorganismen und Zellkulturen (DSZM). Cells were maintained in RPMI 1640 (Lonza) supplemented with 10% fetal calf serum (Lonza) and 1% antibiotic–antimycotic (Lonza). Cells were treated with compounds at the indicated concentrations in exponential growth phase. Proliferation and viability were assessed by trypan blue exclusion analysis at the indicated time points.

## Results and Discussion

For the synthesis of the quinazolines **4–18**, the 2,4-dichloroquinazoline **19** [26] was treated with 4-amino-1-benzylpiperidine at room temperature providing the 4-substituted intermediate **20**, which underwent C2-chloro displacement at the quinazoline ring with the proper amines at 110°C in a sealed tube to provide the desired 2,4-disubstituted quinazolines (Figure 1C).

Compounds **4–18** were tested against human DNMT1 (hDNMT1) and the C-terminal catalytic domain of human DNMT3A (hDNMT3A) to assess their inhibitory activities. Compound **1** and SGI-1027, a known non-nucleoside DNMT inhibitor [27], [28], were used for comparison purposes.

The majority of the tested compounds, when evaluated against DNMT1, were inactive at 100 µM, with the sole exception of the *N*-(1-benzylpiperidin-4-yl)-2-(pyrrolidin-1-yl)quinazolin-4-amine **10**, that showed a dose-dependent DNMT1 inhibition (% inhibition at 100, 32 and 10 µM: 47±1.7, 18±0.8 and 6.5±0.7, respectively) (Table 1). As expected, **1** displayed moderate DNMT1 inhibiting activity (30%), while SGI-1027 was very potent. Surprisingly, the majority of the synthesized quinazolines (**4**, **6–10**, **13** and **14**) selectively inhibited the catalytic domain of hDNMT3A with an inhibition at 100 µM ranging from 47 to 70% (Table 1). In this last assay, SGI-1027 showed high hDNMT3A inhibition, whereas **1** displayed very low activity.

**Table 1:** Percent of inhibition of **4–18** against hDNMT1 and hDNMT3A.

Compd	% inhibition ± SD				
	hDNMT1	hDNMT3A			
	100 µM	100 µM	32 µM	10 µM	3.2 µM
**4**	0	67±3	55±3	39±2	16±4
**5**	0	30±8	14±3	0	ND†
**6**	0	57±8	33±6	14±6	11±5
**7**	0	57±3	32±3	16±2	7±2
**8**	0	53±3	35±5	14±2	7±1
**9**	0	47±5	14±7	5±2	1±1
**10**	47±1.7*	70±4	43±6	21±8	8±4
**11**	0	42±3	ND	8±2	ND
**12**	0	7±0	ND	ND	ND
**13**	0	62±3	52±5	19±13	8±1
**14**	0	63±4	54±3	40±4	13±2
**15**	0	23±2	ND	ND	ND
**16**	0	37±4	ND	8±3	ND
**17**	0	10±0	ND	ND	ND
**18**	0	8±1	ND	ND	ND
**1**	35±8	12±3	ND	ND	ND
SGI-1027	100±0.1	ND	75±7	72±6	80±4

Values are means of two to five experiments ± standard deviation.

† ND, not determined.

*Percent of inhibition at 32 and 10 µM: 18±0.8 and 6.5±0.7%, respectively.

By comparing chemical structures of active versus inactive quinazolines, it is clear that the C2 position of the quinazoline scaffold should be substituted with a large hydrophobic moiety, typically a diazepane (**4**) or piperazine carrying an aryl (phenyl, pyridyl) ring decreasing its basicity (**13**, **14**), or an azacycloalkane such as piperidine (**6**) eventually carrying a methyl group at C4′ position (**8**), azepane (**7**), or pyrrolidine (**10**) to obtain the highest inhibition. The presence of a heteroatom different from N (*i.e.*, S or O) in the C2-ring decreased the DNMT3A inhibitory activity of the compound (**11**) or abrogated it completely (**12**). Increasing the hydrophilic status of the molecules, either by adding a carbonyl group (**15**) at the C2 substituent or by introducing hydroxy- (**16**) or dimethylamino- (**17**, **18**) alkylamino open side chains at C2 gave scarcely active (**16**) or totally inactive (**15**, **17**, **18**) compounds.

**Figure 2:**
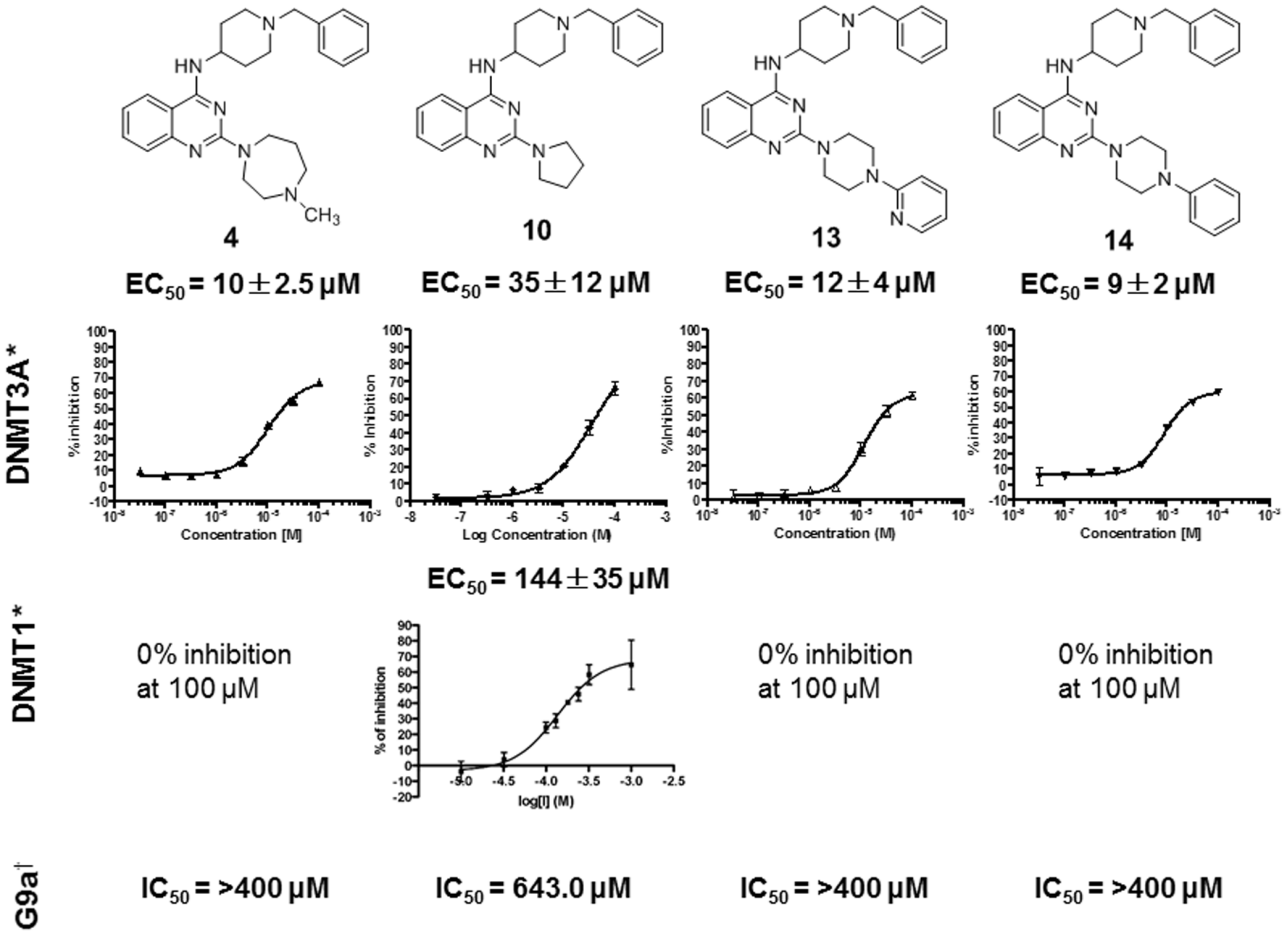
Inhibition values of 4, 10, 13 and 14 against the catalytic domain of hDNMT3A, hDNMT1 and G9a. ^*^Values are means of at least three experiments. ^†^Compounds were tested in a 10-dose IC_50_ mode with 2-fold serial dilution starting at 400 µM. For **4**, **13** and **14** it was no possible to determine IC_50_ values (see File S1).

For compounds **4**, **10**, **13**, and **14**, the most potent against hDNMT3A, the relative EC_50_ values were determined, as well as for **10** against hDNMT1 (Figure 2). In addition, when possible their IC_50_ values against G9a were calculated, to assess their DNMT3A-selectivity and to ascertain if the absence of the C6/C7 dimethoxy substitution effectively abolishes the inhibitory effect of such derivatives against H3K9 methyltransferases (10-dose IC_50_ mode with 2-fold serial dilution starting at 400 µM for G9a inhibition in Table S2 in File S1). Compound **1**, AdoHcy, sinefungin and chaetocin were used as reference drugs (see File S1). Compounds **1**, **4**, **10**, **13** and **14** were also tested against G9a-like protein (GLP), a related H3K9 methyltransferase, to determine the percent of inhibition at 8 µM (see File S1). As expected, among the tested compounds, only **10** was able to inhibit also DNMT1 and G9a, however it being 4- and 18-fold more selective for DNMT3A. The other compounds **4**, **13** and **14** were inactive against DNMT1 (0% of inhibition at 100 µM) and showed >40-fold selectivity for DNMT3A over G9a inhibition (Figure 2). In the G9a assay, the reference compounds showed IC_50_ values from 0.76 to 8.3 µM (see File S1). Against GLP, the tested compounds showed a percent of inhibition ranging from 0 to 10%, whereas the corresponding value for **1** was 91.7% (see File S1).

**Figure 3:**
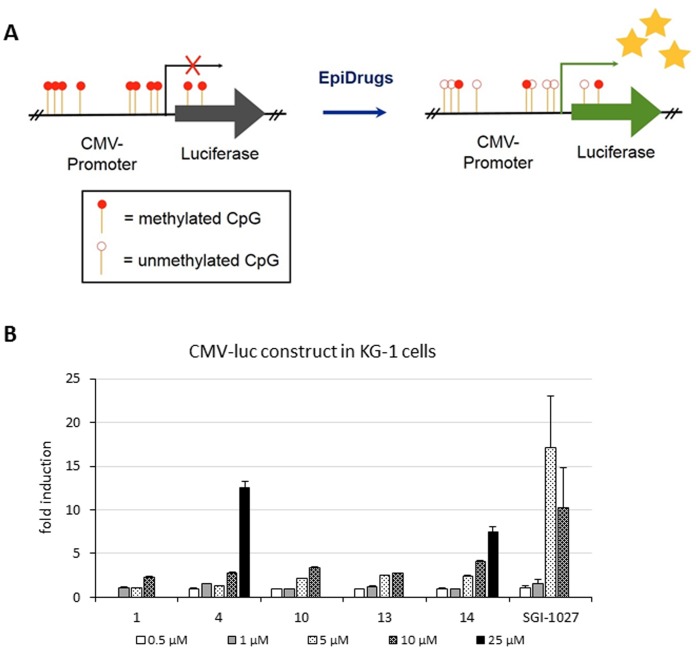
Effect of selected compounds on the induction of the luciferase signal at the CMV-luc construct in KG-1 cells. (A) CMV-luc construct to detect changes in methylation at the CMV promoter in KG-1 cells. (B) Modulation of induction of luciferase expression of CMV-luc after treatment with different doses of **1**, **4**, **10**, **13** and **14**. SGI-1027 was used as a reference drug. Values are means of at least two experiments. Cytotoxicity of DNMT inhibitors in KG-1 cells causes a decrease or loss of luciferase induction.

Compounds **4**, **10**, **13** and **14** were tested in KG-1 cells for their ability to reactivate gene expression, through an integrated luciferase reporter system under the control of a methylated cytomegalovirus (CMV) promoter, which inhibits its expression (CMV-luc assay) [19] (Figure 3A). The structurally related **1** was added for comparison. The fold-induction of the luciferase signal was measured in cells incubated for 24 h in the presence of the drugs at the indicated concentrations, and normalized to the value of the non-treated cells. SGI-1027 was used as a reference drug. At 5 µM the quinazolines **10**, **13** and **14** furnished 2/3-fold induction of the luciferase signal, this effect increasing at 10 µM. At 25 µM **14** showed 7.5-fold increase of the CMV-luc signal, whereas **10** and **13** were not effective because of loss of cells due to the cytotoxicity of the compounds (data not shown). Compound **4** significantly induced the luciferase signal at 10 µM (2.8-fold) and reached its highest value at 25 µM (12.5-fold). Both compounds **4** and **14** at 50 µM were ineffective to reactivate CMV-luc because of cytotoxicity (not shown). SGI-1027 displayed 17-fold of induction of the signal at 5 µM, but at 10 and 25 µM suffered from cytotoxicity giving a decrease to 10-fold and no induction, respectively (Figure 3B).

Molecular modeling studies were performed to explain the reasons behind the hDNMT3A inhibitory activity of the newly discovered analogues of **1**. In particular, the most potent and structurally diverse hDNMT3A inhibitors **4** and **14** were docked into the active site of the enzyme using the Glide software which has been already successfully used to suggest a binding conformation for other DNMT3A inhibitors [29]. To this end, the 2.89 Å resolution X-ray crystal structure of the catalytic hDNMT3A-hDNMT3L tetrameric complex bound to *S*-adenosyl-l-homocysteine (AdoHcy) was used (PDB code 2QRV) [7].

**Figure 4:**
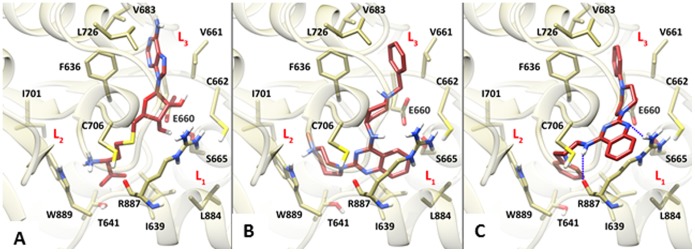
Docking studies of 4 and 14 into the DNMT3A active site. Experimental (A) and theoretical (B and C) binding mode of AdoHcy (A), **4** (B) and **14** (C) in the DNMT3A active site. Protein is depicted as light yellow ribbons and sticks while ligands are depicted as red sticks. Hydrogen-bonds are depicted as dashed blue lines.

Docking simulations achieved for **4** placed the ligand in the enzyme’s binding cleft of AdoHcy with a calculated docking score of −7.232 kcal/mol (Figure 4). In this conformation, the quinazoline core scaffold is embedded in a rather lipophilic and narrow cleft made up by I639, S665, and L884 (herein referred to as L_1_, Figure 4B). Indeed, the limited room available in the aforementioned cleft would not allow hosting of the C6- and C7-methoxy groups of **1** thus explaining why the latter compound is inactive against DNMT3A.

The achieved binding pose also allowed explaining the contribution to ligand binding of the C2- and C4-substituents. In particular, the C2-diazepane moiety takes favorable hydrophobic contact with T641 and I701 residues (herein referred to as L_2_) explaining why substitution of the C2 position with neutral azacycloalkane rings (**6–10**) favorably fills the L_2_ cleft and still results in effective DNMT3A inhibitors. Interestingly, unlike **6–10**, **4** bears a protonated nitrogen (N4 of the diazepane ring) that is able to engage with the adjacent W889 forming a cation-π interaction, which was also detected, through X-ray studies [7], for the terminal positively charged nitrogen atom of AdoHcy in the interaction with DNMT3A (Figure 4A). In agreement with this binding orientation, the absence of hydrophobic and/or cation-π interactions in this region is detrimental for enzyme inhibition (**11**, **12**, **16–18**).

Regarding the C4 substituent, the 1-benzyl-4-piperidinylamino group is optimally oriented to engage a long-range ionic interaction with E660 so that the terminal aromatic ring is able to form a well-oriented T-shaped charge-transfer interaction with F636 and additional hydrophobic contacts in the cleft made up by V661, C662, V683, and L726 (herein referred to as L_3_). Also in this case, the same contacts were already detected for the adenine ring of AdoHcy in its interaction with the enzyme (Figure 4A) [7].

Interestingly, docking results achieved for **14** (docking score −6.353 kcal/mol) placed the ligand in a reverted binding pose in which the C2 and C4 substituents are now lodged in the L_3_ and L_2_ pockets, respectively. Such a binding position places the conformationally rigid C2-phenylpiperazinyl moiety of **14** so as to recapitulate the interaction pattern established by the C4-substituent of **4** and the adenine ring of AdoHcy with the enzyme (Figures 4C, 4B and 4A, respectively). Accordingly, increasing of the steric demands of the C2-substituent (**15–18**) should disrupt the interactions with the L_3_ pocket thus being detrimental for the ligand inhibitory potency. Moreover, the L_2_ pocket is now favorably contacted by the C4-1-benzyl-4-piperidinylamino moiety of **14** through a parallel displaced π-π interaction with W889 aromatic ring. In this position, the above mentioned L_1_ pocket does not seem to be contacted by the ligand quinazoline ring that is now forming a hydrogen-bond between its N1 atom and R887 side chain; the latter residue is also forming an additional hydrogen-bond through its backbone carbonyl oxygen with the ligand exocyclic NH in C4.

To rationalize the lack of inhibition potency of the analogues of **1** against G9a/GLP, compound **4** was docked into the X-ray structure of the GLP enzyme obtained in complex with **1** (PDB code 3FPD) [16]. Glide results indicated that **4** is able to recapitulate the experimental binding position of **1** (Figure S1a in File S1). Interestingly, in the latter compound the C6- and C7- methoxy groups are demonstrated to play a critical role in the ligand inhibition as they provide further interactions to better mimic the substrate/GLP recognition [16] (Figure S1b in File S1), thus explaining the lack of GLP inhibition by **4** in their absence.

Docking studies were also attained for compounds **4** and **14** in the X-ray structure of DNMT1 crystallized in complex with sinefungin (PDB 3SWR, Hashimoto and Cheng, unpublished data), to clarify why these two ligands are inactive against the aforementioned enzyme. Comparison of the DNMT3A and DNMT1 methyltransferase domains reveals that the room available for ligand binding is similar and, for the latter enzyme, the presence of three distinct clefts (L_1_, L_2_ and L_3_) can be also detected. Moreover, experimental data allowed to postulate that, in the case of DNMT1, the inhibitors should interact with the so-called CXXC [28] and autoinhibitory linker [29] domains (residues 646–692 and 699–733, respectively) to induce efficient inhibition. According to Glide calculations, both **4** and **14** should be able to occupy the DNMT1 methyltransferase site (Figure S2a and S2b in File S1). Nevertheless, the residue composition of this region (i.e. negatively charged residues in the L_2_ pocket) should result in low binding affinity. Also, no tight contacts are established with the CXXC and autoinhibitory domains. This should explain why, despite the overall binding site similarities between the two enzymes, **4** and **14** are potent DNMT3A inhibitors without displaying any activity against DNMT1.

**Figure 5:**
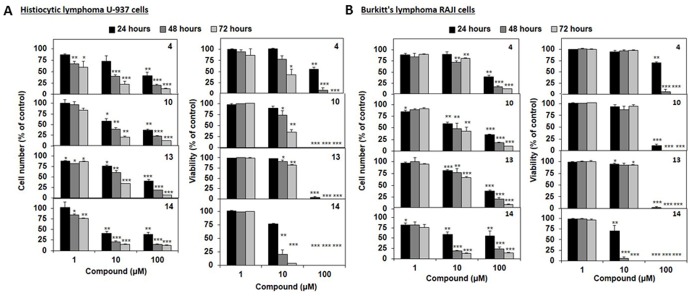
Effects of selected compounds in human lymphoma U-937 and RAJI cells. Cells were treated with the indicated concentration of compounds **4**, **10**, **13** and **14** for up to 72 h. Antiproliferative effects (left) and cell death induction (right) on (A) U-937 and (B) RAJI lymphoma cell lines. Results are the mean ± SD of at least three independent experiments. *, **, *** indicate p<0.05, p<0.01, p<0.005 versus untreated cells, respectively.

Moreover, we investigated the effects of **4**, **10**, **13** and **14** on proliferation and viability of U-937 and RAJI lymphoma cells (Figure 5). Globally, all compounds were cytotoxic in the low micromolar range as they decreased cell proliferation as a consequence of the loss of cell viability. IC_50_ values of **4**, **10**, **13** and **14** on cell viability at 48 h are reported in Table 2. SGI-1027 data [28] have been added for comparison purpose. In agreement with its DNMT3A inhibitory potency, **14** showed the highest antiproliferative effect in the tested cancer cell lines, it being, when compared to SGI-1027, 2.6-fold less efficient against U-937 but 3-fold more potent against RAJI cells.

**Table 2:** Effect of selected quinazoline analogues **4**, **10**, **13** and **14** on human lymphoma U-937 and RAJI cell viability at 48 h*.

compd	IC_50_ (mean ± SD, µM)	
	U-937	RAJI
**4**	16.6±3.8	21.1±2.5
**10**	14.7±3.8	18.7±4.3
**13**	18.8±1.2	19.7±1.4
**14**	4.4±1.4	3.4±0.3
SGI-1027	1.7±1.1	9.1±0.8

*Data represent the mean (± SD) of at least three independent experiments.

## Conclusion

Starting from the quinazolines **1–3**, previously reported as H3K9 methyltransferase/demethylase inhibitors, we explored the possibility to design new related derivatives **4–18** as novel DNMT inhibitors maintaining the C4-substitution typical of **1**, varying the substituent at C2 position and deleting the C6/C7 dimethoxy groups, crucial for the interaction with the H3K9 modifying enzymes. When tested against hDNMT1 and the catalytic domain of hDNMT3A, the majority of **4–18** displayed high potency against DNMT3A and no inhibiting activity against DNMT1, with the only exception of **10**. The most potent compounds against DNMT3A, *i.e.* **4**, **10**, **13** and **14**, were up>40-times selective for DNMT3A over G9a. The same compounds, tested below their cytotoxic dose, increased the luciferase signal of an integrated reporter system in KG-1 cells silenced by a methylated cytomegalovirus (CMV) promoter (CMV-luc assay). In human lymphoma U-937 and RAJI cells, **14** displayed the highest antiproliferative and cell death inducing effects, consistently with the highest inhibitory potency showed against DNMT3A.

## Supporting Information

File S1 **File S1 contains:** Preparation of *N*-(1-benzylpiperidin-4-yl)-2-chloroquinazolin-4-amine (**20**). Chemical and physical data for compounds **4–11**, **13–18**. **Table S1.** Elemental analyses of compounds **4–18**, **20**. **Table S2.** Percentages of G9a inhibition by **4**, **10**, **13** and **14** tested in a 10-dose IC_50_ mode with 2-fold serial dilution starting at 400 µM. IC_50_ graphic data for compound **10**. GLP assay. **Figure S1.** Binding mode of **4** (yellow sticks) in the GLP binding site (green ribbons) as predicted by Glide. **Figure S2.** Predicted binding mode of **4** (a) and **14** (b) in the DNMT1 X-ray structure.
(DOC)
