# Supplementary material for: Properly Substituted Analogues of BIX-01294 Lose Inhibition of G9a Histone Methyltransferase and Gain Selective Anti-DNA Methyltransferase 3A Activity
Source: PLoS One. 2014 May 8;9(5):e96941. doi: 10.1371/journal.pone.0096941 (PMC4014597; doi:10.1371/journal.pone.0096941)
Supplement: File S1 — contains: Preparation of N-(1-benzylpiperidin-4-yl)-2-chloroquinazolin-4-amine (20). Chemical and physical data for compounds 4–11, 13–18. Table S1. Elemental analyses of compounds 4–18, 20. Table S2. Percentages of G9a inhibition by 4, 10, 13 and 14 tested in a 10-dose IC50 mode with 2-fold serial dilution starting at 400 µM. IC50 graphic data for compound 10. GLP assay. Figure S1. Binding mode of 4 (yellow sticks) in the GLP binding site (green ribbons) as predicted by Glide. Figure S2. Predicted binding mode of 4 (a) and 14 (b) in the DNMT1 X-ray structure. (DOC) [file pone.0096941.s001.doc]

D. Rotili, D. Tarantino, B. Marrocco, C. Gros, V. Masson, V. Poughon, F. Ausseil, Y. Chang, D. Labella, S. Cosconati, S. Di Maro, E. Novellino, M. Schnekenburger, C. Grandjenette, C. Bouvy, M. Diederich, X. Cheng, P. B. Arimondo, and A. Mai*

**Properly Substituted Quinazoline Analogues of BIX-01294 Lose Inhibition of G9a Histone Methyltransferase and Gain Selective Anti-DNA Methyltransferase 3A Activity**

**Supporting Information**

**Contents:**

**Chemistry**  S3

**Table S1.** Elemental Analyses of Compounds **4**-**21**, **23** S9

**G9a Assay**  S10

**Table S2.** Percentages of G9a inhibition by **4**, **10**, **13** and **14** tested in a 10-dose IC_50_ mode with 2-fold serial dilution starting at 400 μM S10

**GLP Assay** S11

**Molecular Modelling** S12

**Figure S1.** a) Binding mode of **4** (yellow sticks) in the GLP binding site (green ribbons) as predicted by Glide. b) Superimposition of the experimental binding pose of the dimethylated H3K9 peptide (pink sticks, PDB code 2RFI) and **1** (cyan sticks) in the GLP binding site (green ribbons). S12

**Figure S2.** Predicted binding mode of **4** (a) and **14** (b) in the DNMT1 X-ray structure. S13

**References** S14

**Chemistry**

**Preparation of *N*-(1-benzylpiperidin-4-yl)-2-chloroquinazolin-4-amine (20).** 2,4-Dichloroquinazoline **19** [1] (1 eq, 6.2 mmol, 1.2 g) was dissolved in dry tetrahydrofuran (THF, 50 mL) and 4-amino-1-benzylpiperidine (2.5 eq, 15.4 mmol, 2.9 mL) was added to the clear solution. The mixture was stirred at room temperature and the reaction was completed after 30 min. The resulting white salt in suspension was filtered off and washed with dry THF. The filtrate and the washings were concentrated in vacuum and the crude solid triturated with petroleum ether, collected by filtration, washed with petroleum ether and dried to give **20** as a pure white solid. Mp: 158-160 °C (benzene/cyclohexane). Yield: 86%. ^1^H-NMR (CDCl_3_): δ 1.65-1.69 (m, 2H, C*H*-piperidine ring), 2.14-2.16 (m, 2H, C*H*-piperidine ring), 2.26-2.31 (m, 2H, C*H*-piperidine ring), 2.90-2.93 (m, 2H, C*H*-piperidine ring), 3.58 (s, 2H, C*H_2_*Ph), 4.33 (m, 1H, NHC_4_-*H*-piperidine ring), 5.78 (d, 1H, N*H*), 7.29-7.36 (m, 5H, C*H*-phenyl ring), 7.66-7.68 (t, 1H, C_6_-*H* quinazoline ring), 7.74-7.76 (m, 3H, C_5,7,8_-*H* quinazoline ring). ^13^C-NMR (CDCl_3_) δ 30.3, 51.8, 56.8, 64.6, 114.2, 122.5, 126.6, 127.2, 127.6, 128.4, 128.8, 133.1, 138.6, 150.7, 157.6, 160.3 ppm. HR-MS (ESI) calculated for C_20_H_22_ClN_4_ [M + H]^+^, 353.1533; found, 353.1537.

***N*-(1-Benzylpiperidin-4-yl)-2-(4-methyl-1,4-diazepan-1-yl)quinazolin-4-amine (4).** Mp: 96-98 °C (cyclohexane). Yield: 65%. ^1^H-NMR (DMSO) δ 1.63-1.65 (m, 2H, C*H*-piperidine ring), 1.86 (m, 2H, C*H*-diazepan ring), 1.94-1.97 (m, 2H, C*H*-piperidine ring), 2.05 (m, 2H, C*H*-piperidine ring), 2.24 (s, 3H, N-C*H_3_*), 2.43-2.44 (m, 2H C*H*-diazepane ring), 2.58 (m, 2H, C*H*-diazepane ring), 2.86-2.88 (m, 2H, C*H*-piperidine ring), 3.48 (s, 2H, C*H_2_*Ph), 3.76-3.79 (m, 2H, C*H*-diazepane ring), 3.84 (m, 2H, C*H*-diazepane ring), 4.02 (m, 1H, NHC*H*-piperidine), 7.00 (t, 1H, C*H* quinazoline ring ), 7.22-7.27 (m, 2H, C*H* phenyl ring), 7.32-7.36 (m, 4H, C*H* phenyl ring and N*H*), 7.44-7.48 (m, 1H, C*H* quinazoline ring ), 7.51-7.53 (d, 1H, C*H* quinazoline ring), 8.02-8.04 (d, 1H, C*H* quinazoline ring). ^13^C-NMR (DMSO) δ 26.5, 30.2, 46.8, 51.6, 51.9, 56.9, 57.5, 59.2, 61.9, 64.8, 110.4, 123.1, 127.3, 127.7, 127.8, 128.4, 128.8, 132.8, 138.5, 152.1, 160.1, 184.5 ppm. HR-MS (ESI) calculated for C_26_H_35_N_6_ [M + H]^+^, 431.2923; found, 431.2928.

***N*-(1-Benzylpiperidin-4-yl)-2-(4-methylpiperazin-1-yl)quinazolin-4-amine (5).** Mp: 114-118 °C (cyclohexane). Yiled: 68%. ^1^H-NMR (CDCl_3_) δ 1.62-1.64 (m, 2H, C*H*-piperidine ring), 2.11 (m, 2H, C*H*-piperidine ring), 2.22 (m, 2H, C*H*-piperidine ring), 2.34 (s, 3H, N-C*H_3_*), 2.49 (m, 4H, C*H*-piperazine ring), 2.87-2.90 (m, 2H, C*H*-piperidine ring), 3.54 (s, 2H, C*H_2_*Ph), 3.91 (m, 4H, C*H*-piperazine ring), 4.13 (m, 1H, NHC_4_-*H*-piperidine), 5.45 (s, 1H, N*H*), 7.05 (m, 1H, C*H* quinazoline ring), 7.32-7.33 (m, 5H, C*H* phenyl ring), 7.42-7.49 (m, 3H, C*H* quinazoline ring). ^13^C-NMR (CDCl_3_) δ 30.3, 46.6, 51.9, 52.0, 56.8, 57.2, 64.7, 110.3, 123.1, 127.3, 127.6, 127.9, 128.5, 128.8, 132.3, 138.5, 152.8, 160.4, 184.6 ppm. HR-MS (ESI) calculated for C_25_H_33_N_6_ [M + H]^+^, 417.2767; found, 417.2761.

***N*-(1-Benzylpiperidin-4-yl)-2-(piperidin-1-yl)quinazolin-4-amine (6).** Mp: 118-120 °C (cyclohexane). Yield: 61%. ^1^H-NMR (CDCl_3_) δ 1.68-1.72 (m, 8H, C*H*-piperidine rings), 2.14-2.17 (m, 2H, C*H*-piperidine ring), 2.24-2.29 (m, 2H, C*H*-piperidine ring), 2.93 (m, 2H, C*H*-piperidine ring), 3.59 (s, 2H, C*H_2_*Ph), 3.89-3.90 (m, 4H, C*H*-piperidine ring at C_2_), 4.16 (m, 1H, NHC_4_-*H*-piperidine), 5.52 (s, 1H, N*H*), 7.05 (m, 1H, C*H* quinazoline ring), 7.33-7.38 (m, 5H, C-*H* phenyl ring), 7.49-7.53 (m, 3H, C-*H* quinazoline ring). ^13^C-NMR (CDCl_3_) δ 24.5, 25.5, 30.3, 51.8, 54.8, 56.8, 64.8, 110.1, 123.3, 127.3, 127.7, 127.8, 128.5, 128.8, 132.7, 138.9, 152.3, 160.2, 184.5 ppm. HR-MS (ESI) calculated for C_25_H_32_N_5_ [M + H]^+^, 402.2658; found, 402.2664.

**2-(Azepan-1-yl)-*N*-(1-benzylpiperidin-4-yl)quinazolin-4-amine (7).** Mp: 119-122 °C (cyclohexane). Yield: 71%. ^1^H-NMR (CDCl_3_) δ 1.57-1.67 (m, 6H, C*H* azepane and piperidine rings), 1.82 (m, 4H, C*H*-azepane ring), 2.15-2.25 (m, 4H, C*H*-piperidine ring), 2.90-2.93 (m, 2H, C*H-*piperidine ring), 3.57 (s, 2H, C*H_2_*Ph), 3.83-3.85 (m, 4H, C*H* azepane ring), 4.14-4.16 (m, 1H, NHC_4_-*H*-piperidine), 5.40 (s, 1H, N*H*), 7.03 (m, 1H, C-*H* quinazoline ring) , 7.34-7.37 (m, 5H, C*H*-phenyl ring), 7.47-7.51 (m, 3H, C-*H* quinazoline ring). ^13^C-NMR (CDCl_3_) δ 26.8, 28.0, 30.3, 51.9, 54.2, 56.7, 64.7, 110.3, 123.4, 127.3, 127.7, 127.9, 128.7, 128.9, 132.9, 138.6, 152.5, 160.5, 184.3 ppm. HR-MS (ESI) calculated for C_26_H_34_N_5_ [M + H]^+^, 416.2814; found, 416.2819.

***N*-(1-Benzylpiperidin-4-yl)-2-(4-methylpiperidin-1-yl)quinazolin-4-amine (8).** Mp: 106-108 °C (petroleum ether). Yield: 57%. ^1^H-NMR (CDCl_3_) δ 0.97-0.99 (d, 3H, CHC*H_3_*), 1.19-1.23 (m, 2H, C*H*-piperidine ring at C_2_), 1.65-1.76 (m, 5H, C*H*-piperidine rings), 2.15-2.26 (m, 4H, C*H*-piperidine ring), 2.90-2.94 (m, 4H, C*H*-piperidine rings), 3.58 (s, 2H, C*H_2_*Ph), 4.15 (m, 1H, NHC_4_-*H*-piperidine), 4.86-4.89 (m, 2H, C*H*-piperidine ring at C_2_), 5.40 (s, 1H, N*H*), 7.05 (m, 1H, C-*H* quinazoline ring), 7.30-7.37 (m, 5H, C*H*-phenyl ring), 7.46-7.52 (m, 3H, C-*H* quinazoline ring). ^13^C-NMR (CDCl_3_) δ 20.4, 30.2, 32.3, 34.3, 51.9, 52.3, 56.5, 64.9, 110.6, 123.5, 127.6, 127.9, 127.8, 128.4, 128.8, 132.5, 138.6, 152.3, 160.4, 184.7 ppm. HR-MS (ESI) calculated for C_26_H_34_N_5_ [M + H]^+^, 416.2814; found, 416.2808.

***N*-(1-Benzylpiperidin-4-yl)-2-(2-methylpiperidin-1-yl)quinazolin-4-amine (9).** Mp: 128-129 °C (cyclohexane). Yield: 45%. ^1^H-NMR (CDCl_3_) δ 1.22-1.24 (d, 3H, CHC*H_3_*), 1.53-1.54 (m, 2H, C*H*-piperidine ring at C_2_), 1.64-1.77 (m, 6H, C*H* piperidine rings), 2.14-2.27 (m, 4H, C*H*-piperidine ring), 2.91-3.02 (m, 3H, C*H*-piperidine rings), 3.59 (s, 2H, C*H_2_*Ph), 4.15 (m, 1H, NHC_4_-*H*-piperidine), 4.77-4.81 (m, 1H, C*H*-piperidine ring at C_2_), 5.21 (s, 1H, N*H*), 7.06 (m, 1H, C-*H* quinazoline ring), 7.31-7.39 (m, 5H, C-*H* phenyl ring), 7.50-7.52 (m, 3H, C-*H* quinazoline ring). ^13^C-NMR (CDCl_3_) δ 18.6, 23.3, 25.7, 30.2, 33.8, 51.9, 52.3, 56.8, 62.6, 64.5, 110.3, 123.2, 127.0, 127.2, 127.5, 128.1, 128.8, 132.7, 138.8, 152.3, 160.2, 184.5 ppm. HR-MS (ESI) calculated for C_26_H_34_N_5_ [M + H]^+^, 416.2814; found, 416.2818.

***N*-(1-Benzylpiperidin-4-yl)-2-(pyrrolidin-1-yl)quinazolin-4-amine (10).** Mp: 112-115 °C (petroleum ether). Yield: 68%. ^1^H-NMR (CDCl_3_) δ 1.62-1.66 (m, 2H, C*H*-piperidine ring), 1.98-2.00 (m, 4H, C*H*-pyrrolidine ring), 2.17-2.27 (m, 4H, C*H*-piperidine ring), 2.91 (m, 2H, C*H*-piperidine ring), 3.57 (s, 2H, C*H_2_*Ph), 3.67 (m, 4H, C*H*-pyrrolidine ring), 4.19-4.20 (m, 1H, NHC_4_-*H*-piperidine), 5.30 (s, 1H, N*H*), 7.03 (m, 1H, C-*H* quinazoline ring), 7.28 (m, 2H, C*H*-phenyl ring), 7.36 (m, 3H, C*H*-phenyl ring), 7.44-7.50 (m, 3H, C-*H* quinazoline ring). ^13^C-NMR (CDCl_3_) δ 25.5, 30.5, 51.9, 54.5, 56.9, 64.8, 110.6, 123.0, 127.2, 127.6, 127.9, 128.4, 128.6, 132.7, 138.5, 152.4, 160.5, 184.3 ppm. HR-MS (ESI) calculated for C_24_H_30_N_5_ [M + H]^+^, 388.2501; found, 388.2507.

***N*-(1-Benzylpiperidin-4-yl)-2-thiomorpholinoquinazolin-4-amine (11).** Mp: 155-157 °C (cyclohexane). Yield: 59%. ^1^H-NMR (CDCl_3_) δ 1.71-1.73 (m, 2H, C*H*-piperidine ring), 2.15-2.17 (m, 2H, C*H*-piperidine ring), 2.26-2.32 (m, 2H, C*H*-piperidine ring), 2.69-2.71 (m, 4H, C*H*-thiomorpholine ring), 2.95-2.98 (m, 2H, C*H*-piperidine ring), 3.61 (s, 2H, C*H_2_*Ph), 4.14-4.17 (m, 1H, NHC_4_-*H*-piperidine), 4.23-4.25 (m, 4H, C*H*-thiomorpholine ring), 5.53 (s, 1H, N*H*), 7.09-7.12 (m, 1H, C-*H* quinazoline ring), 7.28-7.38 (m, 5H, C*H*-phenyl ring), 7.50-7.54 (m, 3H, C-*H* quinazoline ring). ^13^C-NMR (CDCl_3_) δ 27.7, 30.5, 51.7, 53.6, 56.8, 64.5, 110.7, 123.5, 127.2, 127.7, 127.9, 128.6, 128.8, 132.7, 138.9, 152.3, 160.5, 184.8 ppm. HR-MS (ESI) calculated for C_24_H_30_N_5_S [M + H]^+^, 420.2222; found, 420.2229.

***N*-(1-Benzylpiperidin-4-yl)-2-(4-(pyridin-2-yl)piperazin-1-yl)quinazolin-4-amine (13).** Mp: 98-100 °C (petroleum ether). Yield: 58%. ^1^H-NMR (DMSO) δ 1.65-1.67 (m, 2H, C*H*-piperidine ring), 1.95-1.98 (m, 2H, C*H*-piperidine ring), 2.10-2.13 (m, 2H, C*H*-piperidine ring), 2.87-2.90 (m, 2H, C*H*-piperidine ring), 3.51 (s, 2H, C*H_2_*Ph), 3.57-3.60 (m, 4H, C*H*-piperazine ring), 3.87 (m, 4H, C*H*-piperazine ring), 4.08 (m, 1H, NHC_4_-*H*-piperidine), 6.65-6.67 (t, 1H, C-*H* pyridine ring), 6.87-6.89 (d, 1H, C-*H* pyridine ring), 7.07 (t, 1H, C-*H* quinazoline ring), 7.29-7.33 (m, 6H, C*H*-phenyl ring and N*H*), 7.53 (m, 2H, C-*H* quinazoline and pyridine rings), 7.63-7.64 (d, 1H, C-*H* quinazoline ring), 8.07-8.09 (d, 1H, C_6_-*H* pyridine ring), 8.13-8.14 (d, 1H, C-*H* quinazoline ring). ^13^C-NMR (DMSO) δ 30.3, 48.5, 49.5, 51.9, 56.8, 64.8, 106.2, 110.3, 117.9, 123.3, 127.3, 127.7, 127.8, 128.4, 128.8, 132.6, 138.2, 138.7, 148.1, 152.3, 158.3, 160.2, 184.3 ppm. HR-MS (ESI) calculated for C_29_H_34_N_7_ [M + H]^+^, 480.2876; found, 480.2871.

***N*-(1-Benzylpiperidin-4-yl)-2-(4-phenylpiperazin-1-yl)quinazolin-4-amine (14).** Mp: 143-145 °C (cyclohexane). Yield: 63%. ^1^H-NMR (CDCl_3_) δ 1.68-1.71 (m, 2H, C*H*-piperidine ring), 2.17-2.20 (m, 2H, C*H*-piperidine ring), 2.26-2.32 (m, 2H, C*H*-piperidine ring), 2.94-2.97 (m, 2H, C*H*-piperidine ring), 3.30 (m, 4H, C*H*-piperazine ring), 3.61 (s, 2H, C*H_2_*Ph), 4.08 (m, 4H, C*H*-piperazine ring), 4.21 (m, 1H, NHC_4_-*H*-piperidine), 5.45 (s, 1H, N*H*), 6.89-6.93 (m, 1H, C-*H* phenyl ring), 7.01-7.03 (m, 2H, C-*H* phenyl ring), 7.11 (m, 1H, C-*H* quinazoline ring), 7.28-7.41 (m, 7H, C-*H* phenyl rings), 7.49-7.54 (m, 3H, C-*H* quinazoline ring). ^13^C-NMR (CDCl_3_) δ 30.3, 49.4, 51.9, 56.6, 64.7, 110.5, 114.7, 121.6, 123.1, 127.2, 127.7, 127.9, 128.5, 128.8, 129.5, 132.7, 138.6, 149.8, 152.5, 160.2, 184.7 ppm. HR-MS (ESI) calculated for C_30_H_35_N_6_ [M + H]^+^, 479.2923; found, 479.2928.

***N*-(1-Benzylpiperidin-4-yl)-2-(4-benzoylpiperazin-1-yl)quinazolin-4-amine (15).** Mp: 213-215 °C (acetonitrile). Yield: 50%. ^1^H-NMR (DMSO) δ 1.63-1.65 (m, 2H, C*H*-piperidine ring), 1.90-1.93 (m, 2H, C*H*-piperidine ring), 2.07 (m, 2H, C*H*-piperidine ring), 2.83-2.86 (m, 2H, C*H*-piperidine ring), 3.32 (m, 2H, C*H*-piperazine ring), 3.47 (s, 2H, C*H_2_*Ph), 3.69-3.78 (m, 6H, C*H*-piperazine ring), 4.05 (m, 1H, NHC_4_-*H*-piperidine), 7.08 (m, 1H, C-*H* quinazoline ring), 7.24-7.32 (m, 6H, C*H*-phenyl ring and N*H*), 7.45-7.52 (m, 6H, C*H*-phenyl and quinazoline rings), 7.64-7.66 (m, 1H, C-*H* quinazoline ring), 8.07-8.09 (d, 1H, C-*H* quinazoline ring). ^13^C-NMR (DMSO) δ 30.5, 50.1, 51.3, 51.9, 56.8, 64.9, 110.7, 123.0, 127.2, 127.3, 127.7, 127.8, 128.4, 128.5, 128.8, 129.5, 132.6, 135.2, 138.6, 152.5, 160.3, 168.9, 184.5 ppm. HR-MS (ESI) calculated for C_31_H_35_N_6_O [M + H]^+^, 507.2872; found, 507.2879.

***N*-(1-Benzylpiperidin-4-yl)-2-(ethanolamino)quinazolin-4-amine (16).** Mp: 116-118 °C (petroleum ether). Yield: 61%. ^1^H-NMR (CDCl_3_) δ 1.60-1.65 (m, 2H, C*H*-piperidine ring), 2.09-2.12 (m, 2H, C*H*-piperidine ring), 2.20-2.23 (m, 2H, C*H*-piperidine ring), 2.89-2.92 (m, 2H, C*H*-piperidine ring), 3.52 (s, 2H, C*H_2_*Ph), 3.62-3.73 (m, 2H, NHC*H_2_*CH_2_OH), 3.87 (t, 2H, NHCH_2_C*H_2_*OH), 4.16 (m, 1H, NHC-*H*-piperidine), 5.37-5.42 (s, 1H, N*H*), 5.48-5.51 (s, 1H, N*H*CH_2_CH_2_OH), 7.10-7.12 (m, 1H, C-*H* quinazoline ring), 7.30-7.36 (m, 5H, C*H*-phenyl ring), 7.50-7.56 (m, 3H, C-*H* quinazoline ring). ^13^C-NMR (CDCl_3_) δ 30.1, 46.0, 51.7, 56.8, 61.1, 64.5, 109.2, 121.3, 125.2, 126.1, 127.0, 128.4, 128.6, 131.0, 138.3, 149.8, 158.5, 177.7 ppm. HR-MS (ESI) calculated for C_22_H_28_N_5_O [M + H]^+^, 378.2294; found, 378.2299.

***N^4^*-(1-Benzylpiperidin-4-yl)-*N^2^*-(2-(dimethylamino)ethyl)quinazoline-2,4-diamine (17).** Mp: 120-122 °C (cyclohexane). Yield: 51%. ^1^H-NMR (DMSO) δ 1.63-1.69 (m, 2H, C*H*-piperidine ring), 1.88 (m, 2H, C*H*-piperidine ring), 2.01-2.07 (m, 2H, C*H*-piperidine ring), 2.13-2.17 (s, 6H, N(C*H_3_*)*_2_*), 2.38-2.42 (t, 2H, NHCH_2_C*H_2_*N(CH_3_)_2_), 2.85-2.88 (m, 2H, C*H*-piperidine ring), 3.30-3.34 (t, 2H, NHC*H*_2_CH*_2_*N(CH_3_)_2_), 3.42 (s, 2H, C*H_2_*Ph), 4.17-4.18 (m, 1H, NHC_4_-*H*-piperidine), 6.21-6.24 (s, 1H, N*H*CH_2_CH_2_N(CH_3_)_2_), 6.98-7.01 (m, 1H, C-*H* quinazoline ring), 7.32-7.39 (m, 6H, C-*H* phenyl ring and N*H*), 7.44-7.47 (m, 2H, C-*H* quinazoline ring), 8.00-8.03 (m, 1H, C-*H* quinazoline ring). ^13^C-NMR (DMSO) δ 30.0, 46.6, 46.7, 51.7, 56.8, 60.5, 64.5, 109.2, 121.4, 125.2, 126.1, 127.0, 128.2, 128.5, 131.0, 138.3, 149.8, 158.5, 177.6 ppm. HR-MS (ESI) calculated for C_24_H_33_N_6_ [M + H]^+^, 405.2767; found, 405.2761.

***N^4^*-(1-Benzylpiperidin-4-yl)-*N^2^*-(3-(dimethylamino)propyl)quinazoline-2,4-diamine (18).** Oil. Yield: 59%. ^1^H-NMR (CDCl_3_) δ 1.64-1.66 (m, 2H, C*H_2_*CH_2_N(CH_3_)_2_), 1.82 (m, 2H, C*H*-piperidine ring), 2.10-2.14 (m, 2H, C*H*-piperidine ring), 2.20-2.267 (m, 8H, NHCH_2_CH_2_CH_2_N(C*H_3_*)*_2_* and C*H*-piperidine ring), 2.40-2.44 (m, 4H, CH_2_C*H_2_*N(CH_3_)_2_ and C*H*-piperidine ring), 2.90-2.93 (m, 2H, C*H*-piperidine ring), 3.53-3.57 (m, 4H, C*H_2_*Ph and NHC*H_2_*CH_2_CH_2_N(CH_3_)_2_), 4.19-4.22 (m, 1H, NHC_4_-*H*-piperidine), 5.51 (s, 1H, N*H*), 7.10-7.12 (m, 1H, C-*H* quinazoline ring), 7.30-7.36 (m, 5H, C-*H* phenyl ring), 7.48-7.54 (m, 3H, C-*H* quinazoline ring). ^13^C-NMR (CDCl_3_) δ 26.5, 30.3, 40.2, 46.5, 51.8, 55.3, 56.4, 64.7, 109.3, 121.3, 125.4, 126.1, 127.0, 128.2, 128.7, 131.3, 138.1, 149.8, 158.4, 177.5 ppm. HR-MS (ESI) calculated for C_25_H_35_N_6_ [M + H]^+^, 419.2923; found, 419.2928.

**Table S1.** Elemental analyses of compounds **4**-**18**, **20**.

| Compd | MW | Calculated, % | | | Found, % | | |
| --- | --- | --- | --- | --- | --- | --- | --- |
|  |  | C | H | N | C | H | N |
| **4** | 430.59 | 72.52 | 7.96 |  | 72.74 | 8.01 |  |
| **5** | 416.56 | 72.08 | 7.74 |  | 71.85 | 7.69 |  |
| **6** | 401.55 | 74.78 | 7.78 |  | 74.65 | 7.82 |  |
| **7** | 415.57 | 75.14 | 8.00 |  | 75.09 | 7.93 |  |
| **8** | 415.57 | 75.14 | 8.00 |  | 75.22 | 8.08 |  |
| **9** | 415.57 | 75.14 | 8.00 |  | 74.94 | 7.97 |  |
| **10** | 387.52 | 74.38 | 7.54 |  | 74.44 | 7.62 |  |
| **11** | 419.59 | 68.70 | 6.97 | 16.69 | 68.52 | 6.88 | 17.02 |
| **12** | 403.52 | 71.44 | 7.24 | 17.36 | 71.66 | 7.32 | 17.14 |
| **13** | 479.62 | 72.62 | 6.94 |  | 72.86 | 7.05 |  |
| **14** | 478.63 | 75.28 | 7.16 |  | 75.08 | 7.11 |  |
| **15** | 506.64 | 73.49 | 6.76 | 16.59 | 73.60 | 6.84 | 16.44 |
| **16** | 377.48 | 70.00 | 7.21 | 18.55 | 69.88 | 7.16 | 18.79 |
| **17** | 404.55 | 71.25 | 7.97 |  | 71.49 | 8.01 |  |
| **18** | 418.58 | 71.74 | 8.19 |  | 71.59 | 8.09 |  |
| **20** | 352.86 | 68.08 | 6.00 | 15.88 | 67.92 | 5.95 | 16.02 |

**G9a Assay**

**Table S2.** Percentages of G9a inhibition by **4**, **10**, **13** and **14** tested in a 10-dose IC_50_ mode with 2-fold serial dilution starting at 400 μM.

| conc., μM | % G9a inhibition | | | |
| --- | --- | --- | --- | --- |
|  | **4** | **10** | **13** | **14** |
| DMSO | 0 | 0 | 3.2 | 5.1 |
| 0.78 | 0 | 0.3 | 9.4 | 17.3 |
| 1.56 | 0 | 13.4 | 21.6 | 13.5 |
| 3.1 | 1.9 | 14.7 | 11.0 | 0 |
| 6.2 | 0 | 4.6 | 16.7 | 4.2 |
| 12.5 | 0 | 5.9 | 0 | 21.3 |
| 25.0 | 0 | 13.0 | 11.1 | 10.6 |
| 50.0 | 7.9 | 18.6 | 27.2 | 16.5 |
| 100.0 | 21.8 | 20.8 | 24.9 | 22.4 |
| 200.0 | 16.4 | 49.5 | 29.2 | 23.8 |
| 400.0 | 23.5 | 37.8 | 14.0 | 24.0 |

Lab codes: **4**, MC2284; **10**, MC2690; **13**, MC2691; **14**, MC2705

Only for compound **10** (MC2690) it was possible to calculate the IC_50_ value and a curve for G9a inhibition:

Reference drugs:

| compd | IC_50_, μM | AdoHcy |
| --- | --- | --- |
| BIX-01294 | 5.3 |
| *S*-adenosyl-l-homocysteine (AdoHcy) | 0.76 |
| sinefungin | 8.3 |
| chaetocin | 8.0 |

The percent of inhibition of another H3K9 methyltransferase, GLP (G9a-like protein), by **4**, **10**, **13** and **14** at 8 μM was determined.

**GLP Assay.** Recombinant human G9a-like protein (GLP) C-terminal fragment containing both the ankyrin repeats and catalytic SET domain (residues 734-1235; pXC758) were purified as described [2]. For histone methylation inhibition, the assay was performed in 20 μL reaction containing 4.6 mM [methyl-^3^H]-AdoMet, 50 μg/mL histone from calf thymus (SIGMA), 10 μg/mL (0.17 μM) GLP, 100 mM KCl, 5 mM (DTT) and 50 mM Tris-HCl pH 8.5. GLP was pre-incubated with AdoMet and 8 µM inhibitors for 5 min at 30 ^o^C before the addition of the histone substrate. After incubation (5 min), the reaction was terminated by the addition of 20% trichloroacetic acid (TCA, Fisher Scientific). The reaction mixture was spotted on GF/A paper circles (Whatman), washed three times with 3 mL of 10% TCA and once with 3 mL of ethanol. The dried circles were subjected to liquid-scintillation counting with Cytoscint scintillant.

*Percent of GLP inhibition at 8 μM:*

**4**: 0%; **10**: 9.2%; **13**: 3.3%; **14**: 10% **1** (BIX-01294, reference compound): 91.7%

**Molecular Modelling**


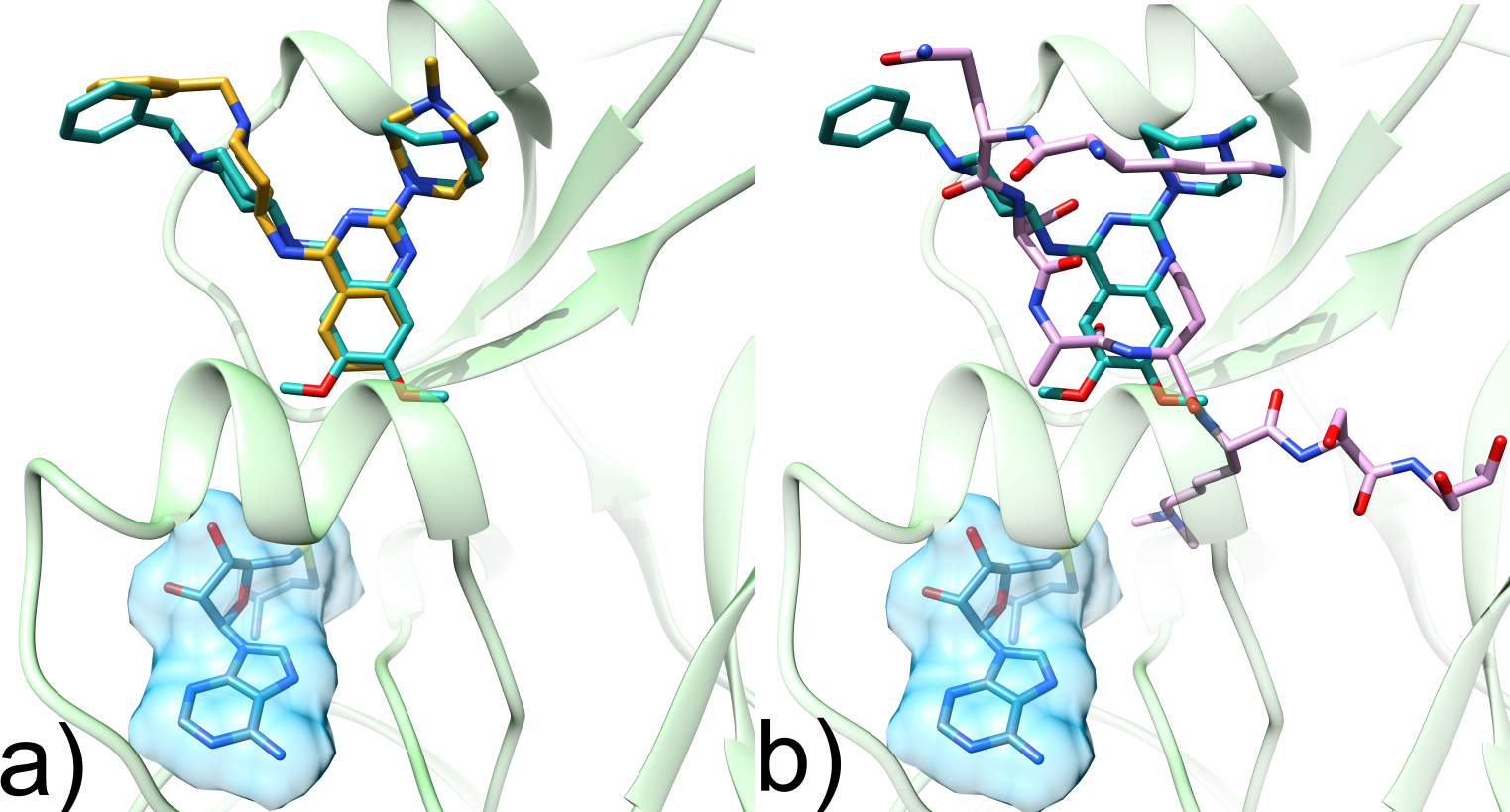


**Figure S1.** a) Binding mode of **4** (yellow sticks) in the GLP binding site (green ribbons) as predicted by Glide. The co-crystal ligand **1** (cyan sticks) was also reported for comparison as well as the co-substrate analogue *S*-adenosyl-l-homocysteine (blue sticks and surface). b) Superimposition of the experimental binding pose of the dimethylated H3K9 peptide (pink sticks, PDB code 2RFI) [3] and **1** (cyan sticks) in the GLP binding site (green ribbons).


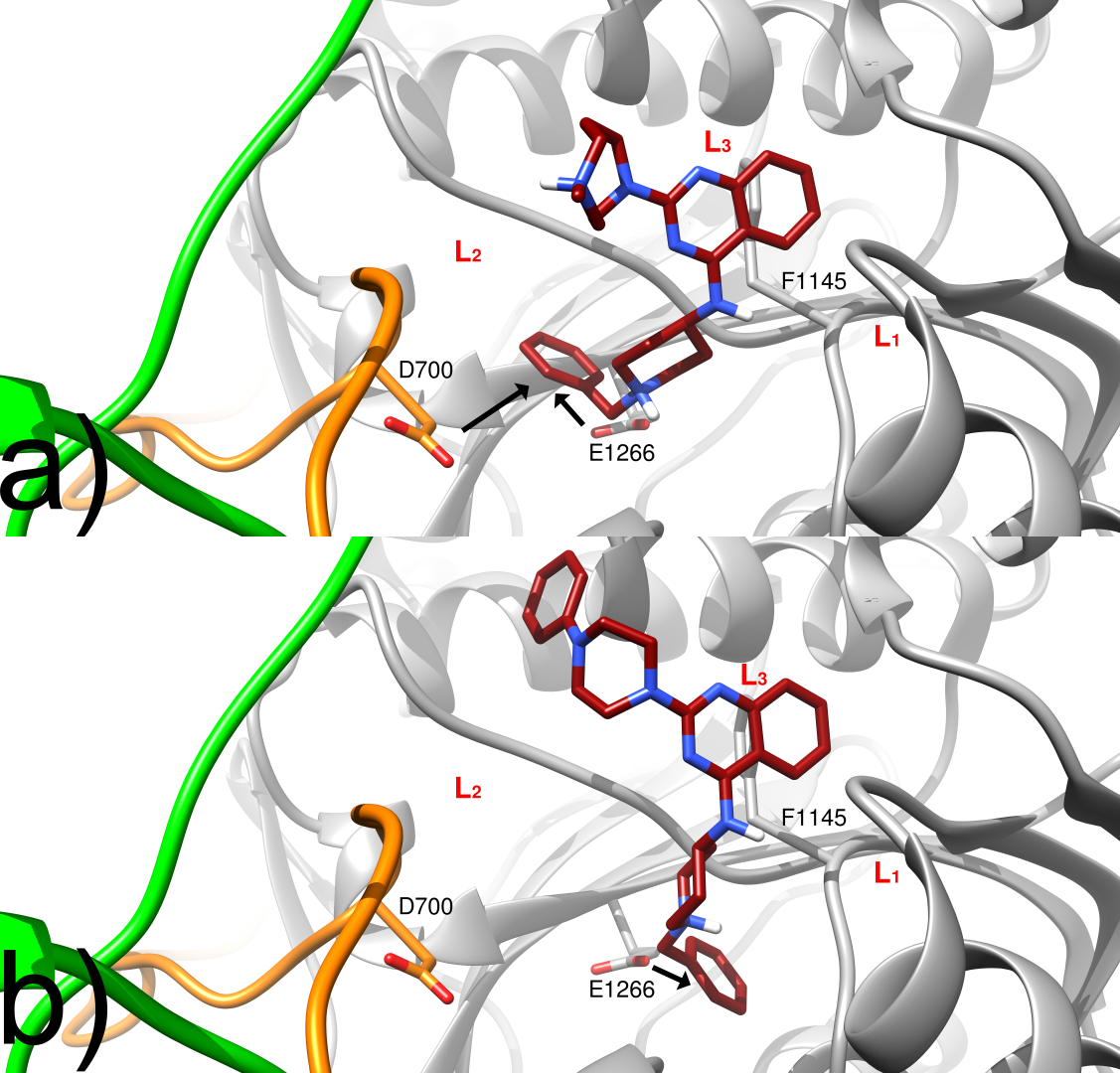


**Figure S2.** Predicted binding mode of **4** (a) and **14** (b) in the DNMT1 X-ray structure. Ligands are represented as red sticks while the enzyme is represented as gray sticks and ribbons with the exception of CXXC and autoinhibitory domains which are represented as green and orange sticks and ribbons, respectively. Black arrows indicate unfavorable contacts between the ligand benzyl group and the negatively charged residues in the enzyme methyltransferase site (L_2_ pocket).

**References**

[1] DeRuiter, J.; Brubaker, A. N.; Millen, J.; Riley, T. N., Design and synthesis of 2-(arylamino)-4(3*H*)-quinazolinones as novel inhibitors of rat lens aldose reductase. *J. Med. Chem.* **1986,** *29* (5), 627-9.

[2] Chang, Y.; Zhang, X.; Horton, J. R.; Upadhyay, A. K.; Spannhoff, A.; Liu, J.; Snyder, J. P.; Bedford, M. T.; Cheng, X., Structural basis for G9a-like protein lysine methyltransferase inhibition by BIX-01294. *Nat. Struct. Mol. Biol.* **2009,** *16* (3), 312-7.

[3] Wu, H.; Min, J.; Lunin, V.V.; Antoshenko, T.; Dombrovski, L.; Zeng, H.; Allali-Hassani, A.; Campagna-Slater, V.; Vedadi, M.; Arrowsmith, C.H.; Plotnikov, A.N.; Schapira, M., Structural biology of human H3K9 methyltransferases. *PLoS One* **2010**, *5*, e8570.
